# BKPyV—Co-Architect of the Fate of a Renal Transplant During a One-Year Observation Period

**DOI:** 10.3390/ijms27062832

**Published:** 2026-03-20

**Authors:** Jacek Furmaga, Marek Kowalczyk, Olga Furmaga-Rokou, Christos A. Rokos, Tomasz Zapolski, Agnieszka Styczeń, Anna Iwan, Dominika Matera, Beata Ewa Chrapko, Leszek Krakowski, Andrzej Jakubczak

**Affiliations:** 1 Department of General and Transplant Surgery and Nutritional Treatment, Medical University of Lublin, 20-954 Lublin, Poland; jacekf61@tlen.pl; 2 Institute of Biological Basis of Animal Production, Faculty of Animal Sciences and Bioeconomy, University of Life Sciences in Lublin, 20-950 Lublin, Poland; marek.kowalczyk@up.lublin.pl; 3 Asklipios Diagnostic Centre, A. Papandreou 204, 56532 Thessaloniki, Greece; 4 Aristoteleio Dental Centre, Akadimou 113, 56224 Thessaloniki, Greece; christos.rokos@gmail.com; 5 Department of Cardiology, Medical University of Lublin, 20-954 Lublin, Poland; zapolia@wp.pl (T.Z.); styczen.agnieszka@gmail.com (A.S.); 6 Department of Gynecological Oncology with a Brachytherapy Subunit, Center of Oncology of the Lublin Region St. John of Dukla, 20-090 Lublin, Poland; anka_i@interia.pl; 7 Department of Neonatology, Pathology and Neonatal Intensive Care, Stefan Cardinal Wyszynski Provincial Specialist Hospital in Lublin, 20-718 Lublin, Poland; doma40@wp.pl; 8 Department of Radiology and Nuclear Medicine, Medical University of Lublin, 20-954 Lublin, Poland; beata.chrapko@wp.pl; 9 Department and Clinic of Animal Reproduction, Faculty of Veterinary Medicine, University of Life Sciences, Gleboka 30, 20-612 Lublin, Poland; leszek.krakowski@up.edu.pl

**Keywords:** BKPyV, RTx, renal transplantation, genotypes, qPCR

## Abstract

To identify BKPyV, the VP1 protein sequence was analyzed and classified into genotypes in 246 RTRs before and after RTx from deceased donors during a one-year observation period. Quantitative assessment of BKPyV was conducted via qPCR. Prior to RTx, genotypes I and IV were identified in the urine (7.27 × 10^6^; 1.20 × 10^5^) and in serum (5.75 × 10^4^; 1.12 × 10^4^). After RTx, genotype I was predominant; identification of DNAuria-BKPyV (62.07%) and BKPyV-DNAemia (55.56%) peaked after three months, and the highest DNAuria-BKPyV titer was also observed after three months (6.48 × 10^9^), whereas the BKPyV-DNAemia titer did not peak until after six months (2.21 × 10^7^). The highest number of copies of genotype IV in the urine was observed after six months (9.54 × 10^9^), while the highest titer in the serum was not observed until after 12 months (3.88 × 10^6^). DNAuria-BKPyV precedes BKPyV-DNAemia, affects a larger group of patients, and has a greater and more easily detected viral load, which makes it not only an earlier marker, but the key predictive marker of greater clinical value than later detection of BKPyV-DNAemia alone. Early monitoring of DNAuria-BKPyV should be the basis of classical screening, and not merely an addition to it, and therapeutic interventions should be undertaken early to prevent nephropathy.

## 1. Introduction

BK polyomavirus (BKPyV) is widespread in the population; antibodies were found to be present in about 90% of the individuals examined [1,2,3], and the DNAuria-BKPyV detection rate ranges from 1% to more than 40% [4,5,6,7].

After the initial infection, the pathogen enters a state of life-long latency in immunocompetent individuals [8], while in immunocompromised individuals, e.g., recipients of renal transplant (RTx) or hematopoietic stem cell transplant (HSCT), it can be reactivated [9,10], potentially leading to BKPyV-associated nephropathy (BKPyVAN) or hemorrhagic cystitis (HC) [11]. It is believed that the main cause of reactivation and the onset of clinical symptoms in these patients is a decline in immunity resulting from the strength of post-RTx immunosuppressive therapy [12]. BKPyVAN occurs in up to about 10% of renal transplant recipients (RTRs) [13,14,15] and can lead to loss of the graft in 50–80% of cases [7,16,17]. To date, specific antiviral therapies against BKPyV are not available; therefore, early diagnosis of virus reactivation is of great clinical importance [14].

The gold standard in BKPyVAN diagnostics is allograft biopsy [18]; however, as this is a highly invasive procedure, surrogate markers of BKPyVAN and of BKPyV infection have been proposed. One of these is qPCR, which can be used to quantify the viral load in serum and/or urine (monitoring of BKPyV-DNAemia and/or DNAuria-BKPyV, respectively). Screening for BKPyV replication via qPCR is the basic strategy for early prediction of the onset of BKPyVAN [19]. Owing to the development of molecular methods, the determination of the viral load in serum and/or urine is increasingly accompanied by the determination of the genotype of the virus. Based on single-nucleotide polymorphisms (SNPs) in the BKPyV protein VP1 region from 1650 to 1936 bp, the virus has been divided into genotypes and subtypes [20,21]. The most common BKPyV type worldwide is genotype I, followed by genotype IV [22].

The complexity of the course of BKPyV infection, due in part to infections with different genotypes of the virus, has prompted the search for new markers or diagnostic methods that would make it possible to reduce the risk of BKPyVAN [23]. One promising direction of research is the identification of screening markers, based on routinely determined parameters that could be used as early predictors of infection prior to further BKPyV diagnostics.

The aim of the study was to determine the incidence and severity of BKPyV infections in a population of dialysis patients subsequently treated by RTx from deceased donors and to determine the effect of the BKPyV genotypes on the function and survival/fate of the graft and the clinical condition of the recipient over a one-year observation period. Clinical parameters and laboratory test results were analyzed, and on this basis, a modification of current diagnostic procedures was proposed and discussed.

## 2. Results

### 2.1. Results Before RTx (Testing Period A0)—Genotypes

Preoperative BKPyV-DNAemia was detected in 3.25% and DNAuria-BKPyV in 46.34% of RTRs (Table 1). In preoperative BKPyV-DNAemia-positive patients, the isolated DNA was classified as genotypes I and IV. Co-infection with both genotypes was found in one patient (n = 1/8; 12.5%). No other BKPyV genotypes were detected in serum.

In the DNAuria-BKPyV-positive group, the isolates belonged to genotypes I and IV, with a few cases of coinfection detected (n = 5/114) (Table 2). As previously described and submitted to the GenBank [4], it was again confirmed that in Poland, among genotype I subtype Ib-2 isolates, variants Ib-2_POL_K (G1809A—OM179842.1) and Ib-2_POL_F (G1809C—OM179843.1) were detected, with their frequency in urine being 58.5% and 41.5%, respectively.

The virus titer in the whole group of BKPyV-DNAemia-positive recipients prior to RTx amounted to 3.1 × 10^4^ and was higher in patients with genotype I (only variant Ib-2_POL_K was present) than in those with genotype IV. In the group of DNAuria-BKPyV-positive patients, the virus titer was 4.21 × 10^6^, and it similarly showed higher values in patients with genotype I than in those with genotype IV. Among variants of subtype Ib-2, the titer in urine was higher for variant Ib-2_POL_K than for Ib-2_POL_F. Only in one patient did the viral load in urine for variant Ib-2_POL_K (4.7 × 10^8^) exceed 1 × 10^7^.

### 2.2. Results Before RTx (Testing Period A0)—Laboratory Tests

Only the hematological parameters (Hct, Hb, and RBC) were significantly reduced in the BKPyV-DNAemia-positive groups compared with the negative group (*p* < 0.05), whereas the remaining pre-transplant clinical and laboratory parameters did not differ significantly among any of the evaluated RTR groups: with or without DNAuria-BKPyV, with or without BKPyV-DNAemia, or between patients with high-level BKPyV-DNAemia (>1 × 10^4^) and BKPyV-DNAemia-negative recipients (Table 2, Table 3 and Table 4).

The small size of the BKPyV-DNAemia-positive and BKPyV-DNAemia (>1 × 10^4^) groups did not permit further statistical analysis. In the DNAuria-BKPyV-positive group, Hb and CRP values were significantly lower in patients with genotype IV than those in patients with genotype I. No other differences among the parameters and laboratory tests conducted were observed (Table 5).

The collection of 24 h urine in the BKPyV-DNAemia-negative group recorded slightly higher values than those in both the BKPyV-DNAemia-positive and the BKPyV-DNAemia patients (>1 × 10^4^). It was also measured higher in the DNAuria-BKPyV-negative group than in the DNAuria-BKPyV-positive recipients, in patients with the detected genotype I compared to IV (Table 5 and Table S3), and in RTRs with variant Ib-2_POL_K than in those with Ib-2_POL_F (*p* = NS).

### 2.3. Results After RTx (Testing Periods Aw–D)—Genotypes

Detection of infection in urine peaked after 3 months (50.58%) post transplantation, followed by a decrease to 46.43% at 12 months, a value comparable to the result prior to RTx (46.34%) (Table 6). The proportion of BKPyV-DNAemia-positive recipients showed a similar pattern peaking at 3 months (26.16%) and a later decrease by the end of the observation period (19.61%) (Table 7). Among both DNAuria-BKPyV-positive and BKPyV-DNAemia-positive patients, only two BKPyV genotypes were identified (I and IV). Simultaneous coinfection of these genotypes was detected in only a few patients.

The prevalence of genotypes also varied during the one-year observation period. Post-RTx, genotype I was identified in 56.31–62.07% of DNAuria-BKPyV-positive patients and dominated over genotype IV in each testing period (38.83–33.33%). Similarly, in BKPyV-DNAemia-positive recipients, continual predominance of genotype I was observed; its frequency increased up to 55.55% after three months and decreased to 45.46% by the end of the observation period. The prevalence of genotype IV post-RTx oscillated between 35.55 and 42.42%, and the highest values were observed at the end of the observation period.

Among BKPyV-DNA isolates of genotype I subtype Ib-2 in DNAuria-BKPyV-positive patients, variant Ib-2_POL_K was continually dominant over Ib-2_POL_F; it was detected with decreasing frequency (63.79–58.33%), in contrast to Ib-2_POL_F and its peak (41.66%) appearing at the end of the observation period. In serum, the variant Ib-2_POL_K declined to 66.67% at 12 months of observation. In contrast, Ib-2_POL_F was not detected until about one month after RTx, and its detection rate increased steadily until the end of the observation period (33.33%).

**Figure 1:**
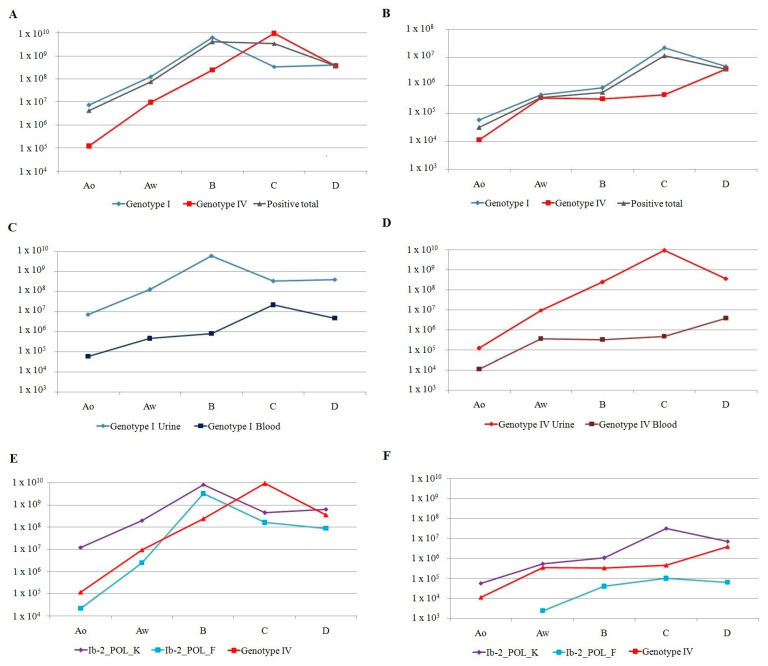
Dynamics of changes in viral load over the one-year observation period, divided into: replication within individual genotypes in urine (**A**) and blood (**B**), replication in urine and blood of genotype I (**C**) and IV (**D**), respectively, and replication within individual variants in urine (**E**) and blood (**F**). A0—before RTx, Aw—1 month after RTx, B—3 months after RTx, C—6 months after RTx, D—12 months after RTx.

The viral load increased rapidly post-RTx in both the DNAuria-BKPyV-positive (Table 8) and BKPyV-DNAemia-positive groups (Table 9). Virus titers were highest in urine after 3 months and in serum after 6 months (4.10 × 10^9^ and 1.15 × 10^7^ copies, respectively), and later decreased slowly by the 12th month. The peak DNAuria-BKPyV and BKPyV-DNAemia titers were reached due to the accumulation resulting from the highest load of genotype I (6.48 × 10^9^ and 2.21 × 10^7^, respectively) and the high but still increasing loads of genotype IV. The highest number of copies of genotype IV was observed at 6 months post-RTx in urine and at 12 months in serum (9.54 × 10^9^ and 3.88 × 10^6^ copies/mL, respectively) (Figure 1A–D).

Both genotype I variants exhibited the highest DNAuria-BKPyV titers at 3 months and the highest BKPyV-DNAemia titers at 6 months post-RTx. In all testing periods, the variant Ib-2_POL_K was dominant over Ib-2_POL_F (Figure 1E,F).

### 2.4. Results After RTx (Testing Periods Aw–D)—Laboratory Tests

Analysis of post-RTx laboratory parameters across DNAuria-BKPyV-positive/-negative and BKPyV-DNAemia-positive/-negative groups shows that, at all observation periods, kidney function was significantly better in virus-negative individuals (Cr-creatinine, eGFR, urea; *p* < 0.01) (Tables S1 and S4). In the BKPyV-DNA-negative groups, the laboratory results for graft function at 12 months of observation were comparable to those obtained in healthy individuals in the general population. In virus-negative patients (in urine and serum), the 24 h urine collection values were higher compared to the BKPyV-DNA-positive groups, and significantly higher after the 6th and 12th month of follow-up (*p* < 0.05). By the end of the observation period, the BKPyV-DNA-negative groups demonstrated statistically significantly higher values of Hb, Hct and RBC (*p* < 0.05), the DNAuria-BKPyV-positive group showed lower WBC, LYM, and PLT values (*p* < 0.05), while in the smaller, BKPyV-DNAemia-positive group, statistical significance was confirmed only for WBC (*p* < 0.05).

In the 12th month of the observation: CRP values even within the acceptable range were found elevated in positive groups (*p* = NS), D-dimer values were significantly higher (*p* < 0.05) compared to the already elevated levels of the negative groups, and RTRs with genotype IV presented higher creatinine levels than those obtained with genotype I (*p* = NS) (Tables S2–S5).

During the entire observation period, only minor differences in 24-hour urine collection values were observed between patients with each genotype (*p* = NS), as well as between genotype I variants Ib-2_POL_K and Ib-2_POL_F (*p* = NS) (Table S3).

In the BKPyV-DNAemia (>1 × 10^4^) group, as expected, graft function parameters showed signs of significant damage, accompanied by lower 24 h urine volume, lower hematological parameters and higher CRP and D-Dimer values in comparison with the BKPyV-DNA-negative group (Table 10 and Table S6).

## 3. Discussion

The results of the study call into question the classical approach according to which BKPyV infection is merely a consequence of the transfer of the infection from the donor and excessive immunosuppression [24]. Our data analysis demonstrated that DNAuria-BKPyV was present in nearly half of patients before RTx (46.3%), and BKPyV-DNAemia in 3.25%, which indicates that infection was already present at the dialysis stage and reactivated once favorable conditions emerged after RTx [7]. Post-RTx, the percentage of BKPyV-DNA-positive recipients showed a rapid increase reaching its highest level at 3 months after transplantation, as detected in both urine (50.58%) and serum (26.16%). Urinary viral load peaked at 3 months post-RTx (4.10 × 10^9^ copies/mL), whereas serum viral load reached its maximum at 6 months (1.15 × 10^7^ copies/mL) and subsequently declined gradually over the remainder of the 12-month observation period. The presented dynamics of infection are in partial agreement with the observation made by Babel et al. [25].

According to the findings of our study, the genotype of the virus seems to be a factor that affects the course of infection, partially determining the fate of the transplanted kidney. Individual genotypes have varying distribution on a global scale, with genotype I appearing predominant. In European countries, there is also a high prevalence of genotype IV [26,27]. Genotype I subtype Ib-2 is predominant in Poland, with variants distinguished on the basis of a nonsynonymous G1809A/C mutation: Ib-2_POL_K and Ib-2_POL_F variants [4], while the isolates listed in bioinformatics databases from various parts of the world are 100% identical to the variants described in the present study. A high rate of genotype IV and coinfection with both genotypes was observed. The prevalence observed in our study is slightly higher than most literature data [24,28,29], but consistent with the results published from another facility in Poland: DNAuria-BKPyV (29.4%), BKV-DNAemia (8.26–25.5%) and BKPyVAN (7.87–14.93%), virus titer 2.3–9.0 log10 copies/mL in serum [30,31,32]. The distribution of individual genotypes in a given population may also represent a unique pattern of endemic evolution of a variant, such as the one occurring in Vietnam following a nonsynonymous mutation at position A1745G, identified in 95% of RTRs [33], or even changed over time [26]. Throughout the entire observation period, the average DNAuria titer exceeded the DNAemia titer by two orders of magnitude, with the sole exception of the values at 3 months post-RTx. DNAuria-BKPyV therefore precedes BKPyV-DNAemia, affects more patients and has a higher and more easily detected viral load. The aforementioned characteristics suggest that it could be used as an earlier marker and, even more importantly, as a key predictive index of high clinical value, related to the later detection of the virus in serum alone.

Prior to RTx, no major difference among the tested parameters (*p* = NS) was observed among the BKPyV-DNA-negative and BKPyV-DNA-positive groups of recipients, as a clear manifestation of viral infection. The only exception was the hematological (Hb, Hct, and RBC) parameters in the BKPyV-DNAemia-positive group. However, it seems that the “clinical silence” prior to the transplant does not imply the absence of risk. Variations in hematological, inflammatory and renal function parameters were observed post-RTx and are directly and causally associated with longer hospitalization (*p* < 0.05). The deterioration of graft function, a decrease in 24 h urine collection and CBC parameters (complete blood count parameters) in the BKPyV-DNA-positive group do not appear to be incidental, but systematically follow the increase in DNAuria-BKPyV and BKPyV-DNAemia, especially in the period from 6 to 12 months after RTx. This can be interpreted as a pathophysiological sequence of events that may demonstrate that the virus co-shapes the trajectory of the graft, influencing the recipients’ immunological and hematological response.

Hematological changes are the result of complex, concurrent processes such as chronic inflammation, rejection, allograft dysfunction, effects of medications (immunosuppressive, antiviral, and antimicrobial) and, obviously, viral infections [34]. Their consequence is subclinical inflammation and a prothrombotic state, which may intensify one another. Elevated CRP is positively correlated with a deterioration in graft function long after transplantation [35], accompanied by a statistically significant increase in D-dimer levels. Moreover, an increase in CRP values is associated with higher mortality rates in RTRs [36] and is considered a predictive factor of coronary events in the case of both ESRD (end-stage renal disease) patients and in the general population [37]. Although this can be treated as a manifestation of the systemic acute phase response, it cannot be ruled out that D-dimers are a marker of thrombosis in the renal microcirculation, which can contribute to graft damage, including in the context of BKPyVAN [38]. High D-dimer levels can exacerbate inflammatory and coagulation processes, leading to acute kidney injury (AKI) [38], and their concentration is directly correlated with creatinine levels and graft function [39].

Hematological complications following transplantation include post-transplant anemia (38–42%) [40,41], leukopenia (20–75%) [30,42], and thrombocytopenia (about 30%) [30], which are most often observed during the first few months after transplantation [40]. They may be due to drug-induced myelosuppression, and since their toxic effect is dose-dependent [40], appropriate, individualized treatment and close monitoring of drug levels remain a key element of treatment. BKPyV replication is also considered responsible for myelosuppression, although this mechanism is not fully understood [43]. It is likely that, like other viruses, BKPyV affects helper and stromal cells, changes the expression of adhesion molecules or directly infects stem cells, impairing hematopoiesis [44].

Complications associated with BKPyV affect both the donor’s kidney and the recipient’s bone marrow [14]. Most likely, both the decrease in hemoglobin levels and the increase in creatinine levels observed in the present study are caused by stromal cell dysfunction and concomitant clinical infection in post-RTx patients. These parameters constitute markers of deteriorating graft function, expressed as a significant decrease in the Hb/Cr ratio. The observed trend of persistent deterioration of renal parameters measured through our observation period suggests that the effect of the damage is long-lasting and that therapeutic interventions should be undertaken early in order to prevent nephropathy [25].

Our study demonstrates that early monitoring of DNAuria-BKPyV should be incorporated into classical screening and not merely as an addition to it. In other words, not only do the effects of immunosuppression allow the virus to develop, but the virus itself actively shapes the recipient’s immune status; therefore, instead of treating BKPyV as a “passenger”, we should perceive it as a co-architect of the outcome of transplantation, graft fate and patient survival. A key question from a clinical and therapeutic perspective is whether individual genotypes or subtypes lead to various manifestations of the disease. This may have potential treatment implications, as knowing the molecular variant could enable more precise, individualized therapy. It is well established that polymorphisms in the nucleotide sequence, especially in the BC loop, can influence traits of the virus, such as tropism for specific cells, the replication rate, or the ability to evade the host’s immune system [4,33,45,46,47,48,49,50].

The appearance of viruria followed by viremia is well known, but our results add to this knowledge, demonstrating that the highest incidence of DNAuria-BKPyV-positive and BKPyV-DNAemia-positive patients occurred three months post-RTx. In this study, we observed that viral-load dynamics are genotype-dependent. In genotype I infection, the viral load peaked at 3 months in urine and at 6 months in serum—a three-month delay. In genotype IV infection, the urinary viral load peaked later, at 6 months, while the serum viral load did not peak until 12 months—a six-month delay relative to urine. Our results indicate that genotype IV attains higher peak levels of DNAuria-BKPyV, replicates later in serum, and has a higher percentage infection rate in serum than in urine, which may be due to more effective evasion of the host’s immune mechanisms. Pastrana et al. [51] demonstrated that genotypes I and IV belong to different serotypes and that while nearly all healthy subjects had BKPyV genotype I neutralizing antibodies, a majority of subjects did not detectably neutralize genotype III or IV.

The more rapid viral load increase observed in both urine and serum in patients infected with genotype I, especially in the initial period post transplantation, seems to be consistent with the findings of studies indicating that BKPyV subtype I replicates more efficiently than BKPyV subtype IV in human renal epithelial cells [52]. The later but more rapid replication of genotype IV and perhaps lower susceptibility to the host’s immune mechanisms may indicate a more chronic type of infection, predispose the patient to significant deterioration of graft function, and translate into a potentially higher risk of BKPyVAN. This seems to be in agreement with the literature, in which infection by genotype IV presents as one of the risk factors for BKPyVAN [24,46,53].

Chronic deterioration of graft function is observed in the case of both genotypes (I and IV) in comparison with the BKPyV-DNA-negative group. Comparison of creatinine, eGFR, and hematological parameters indicated that genotype IV showed less favorable values at the conclusion of the observation period (*p* = NS).

Ultimately, the question is whether we seek only to confirm the presence of BKPyV and its adverse effects, or to more effectively prevent infection progression. The BKPyV-DNAemia (>104) group selected in our study essentially confirms the poorest renal parameter outcomes, accompanied by reduced 24 h urine collection volumes, lower hematological values, and higher inflammatory markers—findings that are consistent with the literature [54]. Classical models of risk assessment for the course of BKPyV infection based on biochemical parameters are no longer sufficient; there is a need for new algorithms taking into account the genetic polymorphism of the virus and the dynamics of its replication. It is no longer only a question of whether a patient has a BKPyV infection; we want to know what genotype is causing it, where and when it is replicating, what the pro-inflammatory and prothrombotic response is, and how it acts together with the immunosuppression profile. Until now, it has been assumed that the conflict following transplantation is between immunosuppression and rejection, but the present study indicates that a third factor—BKPyV infection—should be taken into account as well, in terms of both genetic polymorphism of the virus and its load. Thus, the equation has three components: immunosuppression–rejection–virus. Therefore, genotyping of the virus is necessary in practice. There is also a need to redefine “optimal immunosuppression”—instead of universal, rigid regimens, we should speak of a dynamic balance adjusted to varied replication of the virus. Only a holistic approach and integration of these three axes (genotype–load–immunosuppression) will enable true personalization of post-transplant care.

## 4. Materials and Methods

### 4.1. Study Group

**Figure 2:**
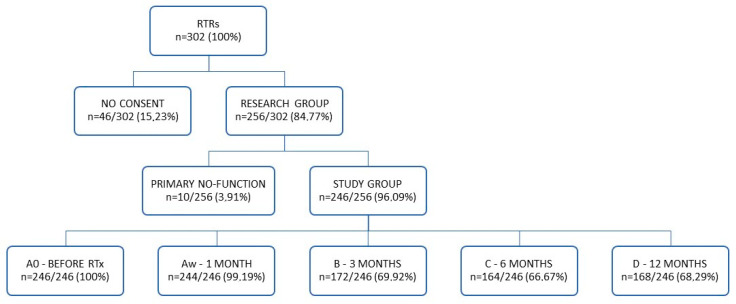
Study design.

The study was carried out from January 2007 to December 2018 in all recipients (n = 302) admitted for RTx to our department (Figure 2). The exclusion criteria were the absence of written consent, non-compliance with follow-up visits, and lack of sufficient biological material for further genetic testing (n = 46/302; 15.23%). Other categories of exclusion were post-transplant RTRs with primary non-function (PNF) and the need for graftectomy (n = 10/256; 3.91%).

Ultimately, the group comprised 246 white adult patients (Table 11). Follow-up periods were established at 1, 3, 6 and 12 months post-RTx. The samples were stored at −80 °C. The study was approved by the Bioethics Committee of the Medical University of Lublin, Poland (KE-0254/29/2007; KE-0254/197/2010; KE-0254/50/2012; KE-0254/259/2015; KE-0254/281/2017).

### 4.2. Treatment Procedure

Immunosuppressive treatment was based on a basic, combined three-drug immunosuppression regimen, consisting of calcineurin inhibitors (CNIs), mycophenolates and steroids (Figures S1–S3). Depending on the immunological risk, some of the patients received either monoclonal or polyclonal antibodies (Basiliximab, ATG, respectively). Post-RTx, we observed primary non-function and delayed graft function (10 and 82 patients, respectively). BKPyVAN was observed in only three cases (Table 12).

### 4.3. BKPyV-DNA Extraction, PCR Amplification, and Genotyping

DNA was extracted from serum and urine with the DNeasy Blood & Tissue Kit (Qiagen, Hilden, Germany) as previously described [4]. After extraction, DNA samples were verified by amplification with primers specific to human β-actin [55] and used to detect BKPyV [4]. The primers used in BKPyV detection amplified a partial sequence of the VP1 protein, spanning from 1630 to 1956 bp in sequence NC_001538 [22].

PCR products were separated in a 2% agarose gel with ethidium bromide. The lengths of the bands were determined by comparing them with GeneRuler 100 bp size markers (Thermo Fisher Scientific, Foster City, CA, USA). The electrophoresis results were analyzed under UV light with Scion Image software (version 4.0.2) (Scion Corporation, Frederick, MD, USA).

Amplification products were subjected to sequencing and bioinformatic analysis as previously described [4]. BKPyV isolates were classified into genotypes based on polymorphic nucleotides proposed by Randhawa et al. [56], using an algorithm designed by Morel et al. [20].

### 4.4. Quantitation of BKPyV-DNA by Real-Time Polymerase Chain Reaction

BKPyV was quantified by qPCR, using the GeneProof BK/JC Virus PCR Kit in an ABI Prism^®^ 7500 Real-Time PCR System (Thermo Fisher Scientific, Waltham, MA, USA) according to the kit manufacturer’s instructions [4]. Viral load was expressed as the number of BKPyV copies per mL. The GeneProof BK/JC Virus PCR Kit differentiates BK virus (BKPyV) from JC virus (JCPyV) by using virus-specific molecular probes labeled with distinct fluorescent dyes. The assay is based on real-time PCR with probes designed to hybridize selectively to target sequences specific for each virus. In this system, the presence of BK virus is indicated by an increase in fluorescence of the FAM fluorophore, while the presence of JC virus is indicated by an increase in fluorescence of the Cy5 fluorophore. Because each probe is labeled with a different fluorescent dye and detected in separate channels of the ABI Prism 7500 Real-Time PCR System (Thermo Fisher Scientific, Waltham, MA, USA), the assay enables simultaneous detection and clear discrimination between BKPyV and JCPyV within the same reaction.

### 4.5. Statistics

All data were analyzed using Statistica 13 software (TIBCO Software Inc., Palo Alto, CA, USA). Normal data distribution was analyzed by Shapiro–Wilk test and variance homogeneity by Levene’s test. To compare non-normal distributed data, Mann–Whitney U and Kruskal–Wallis tests were applied. Normally distributed data were subjected to *t*-test and one-way analysis of variance (ANOVA), followed by Tukey’s (HSD) test, which was used to compare means. Statistical significance was set at *p* < 0.05. Means and standard error of the mean are given in the tables. Categorical data were analyzed by using Fisher’s exact test or Pearson’s χ^2^ test, depending on sample size. All tests were 2-sided, and *p* < 0.05 was considered statistically significant.

## 5. Conclusions

1. High BKPyV-DNAemia and DNAuria-BKPyV are risk factors for graft injury, the development of BKPyVAN and poorer prognosis for the transplanted organ.

2. Without discounting the possibility of transfer of BKPyV infection together with the donor’s organ, our data suggest that the infection is already present at the dialysis stage and is waiting for favorable conditions for further progression, which occur after RTx.

3. DNAuria-BKPyV precedes BKPyV-DNAemia, affects a larger group of patients, and has a greater and more easily detected viral load, which makes it not only an earlier marker, but also a key predictive marker of greater clinical value than later detection of the virus in serum alone.

4. A priori to RTx, a statistically significant difference in hematological values of BKPyV-DNAemia-positive/-negative groups was noted. No other differences in inflammatory, nutritional, renal function parameters or in 24 h urine collection were recorded.

5. Post-RTx, increasingly after 6 months, rising DNAuria-BKPyV and BKPyV-DNAemia are accompanied by worsening signs of graft dysfunction, decreasing 24 h urine collection, and intensifying hematological abnormalities. These findings suggest that the damage is long-lasting and that therapeutic intervention should be initiated early to prevent BKPyVAN.

6. Genotype I is characterized by a rapid onset of viral load (3–6 months), whereas genotype IV shows later replication in the serum, a higher prevalence in serum than in urine, and the highest peak levels of DNAuria-BKPyV, which may be due to more effective evasion of the host’s immune mechanisms.

7. Classical models of risk assessment for the course of BKPyV infection based on biochemical parameters are no longer sufficient; there is a need for new algorithms taking into account the genetic polymorphism of the virus and the dynamics of its replication, the pro-inflammatory and prothrombotic response, and how it acts together with the immunosuppression profile.

8. There is a need to redefine “optimal immunosuppression”. Only a holistic approach comprising an integration of the three axes (genotype–load–immunosuppression) will enable true personalization of post-transplant care.

9. Early monitoring of DNAuria-BKPyV should be incorporated into classical screening and not merely as an addition to it. 

## Figures and Tables

**Table 1:** Genotype and subtype distribution and viral load (copies/mL) in patients with DNAuria-BKPyV and BKPyV-DNAemia in period A0.

Genotype/Variant		BKPyV-DNAemia		DNAuria-BKPyV	
		Frequency—n (%)	Viral Load	Frequency—n (%)	Viral Load
BKPyV-Negative		238 (96.75)	NA	132 (53.66)	NA
BKPyV-Positive		8 (3.25)	3.10 × 10^4^	114 (46.34)	4.21 × 10^6^
Genotype I		3 (37.50)	5.75 × 10^4^	65 (57.02)	7.27 × 10^6^
	Ib-2_POL_K	3 (100)	5.75 × 10^4^	38 (58.46)	1.24 × 10^7^
	Ib-2_POL_F	Non detected	Non detected	27 (41.54)	2.16 × 10^4^
Genotype IV		4 (50.00)	1.12 × 10^4^	44 (38.60)	1.20 × 10^5^
Co-infection		1 (12.50)	3.10 × 10^4^	5 (4.39)	3.49 × 10^5^

**Table 2:** Comparison of the measured parameters between DNAuria-BKPyV-negative and DNAuria-BKPyV-positive groups before RTx in the A0 period.

Parameter	DNAuria-BKPyV-Negative—n = 132	DNAuria-BKPyV-Positive—n = 114	*p*-Value
CRP (mg/L)	2.66 ± 0.24	2.55 ± 0.2	0.933
D-dimer (ng/mL)	790.87 ± 97.33	890.45 ± 138.96	0.819
Na^+^ (mmol/L)	139.58 ± 0.26	139.09 ± 0.32	0.483
K^+^ (mmol/L)	4.77 ± 0.06	4.83 ± 0.06	0.446
Creatinine (mg/dL)	6.79 ± 0.22	7.15 ± 0.24	0.273
eGFR (mL/min/1.73 m^2^)	10.15 ± 0.97	9.19 ± 0.25	0.926
Urea (mg/dL)	81.85 ± 3.27	82.14 ± 3.93	0.791
Hct (%)	35.49 ± 0.39	35.95 ± 0.44	0.441
Hb (g/dL)	11.79 ± 0.13	11.98 ± 0.15	0.368
Hb/Creatinine ratio	1.94 ± 0.06	1.87 ± 0.06	0.561
RBC (cells/μL)	3.84 ± 0.04	3.84 ± 0.05	0.842
WBC (cells/μL)	6.6 ± 0.15	6.81 ± 0.15	0.324
LYMPH (cells/μL)	1.73 ± 0.06	1.58 ± 0.06	0.079
PLT (cells/μL)	221.78 ± 5.8	208.15 ± 5.15	0.150
Albumin (g/dL)	4.32 ± 0.04	4.3 ± 0.05	0.925
Total protein (g/dL)	7.11 ± 0.06	7.1 ± 0.07	0.823
Diuresis (mL/per day)	746.21 ± 67.4	487.28 ± 46.35	0.059

**Table 3:** Comparison of the measured parameters between the BKPyV-DNAemia-negative and BKPyV-DNAemia-positive groups before RTx in the A0 period.

Parameter	BKPyV-DNAemia-Negative n = 238	BKPyV-DNAemia-Positive—n = 8	*p*-Value
CRP (mg/L)	2.62 ± 0.16	2.16 ± 0.66	0.640
D-dimer (ng/mL)	854.95 ± 87.33	356.67 ± 17.37	0.216
Na^+^ (mmol/L)	139.39 ± 0.21	138.25 ± 0.73	0.131
K^+^ (mmol/L)	4.80 ± 0.04	4.84 ± 0.21	0.712
Creatinine (mg/dL)	6.94 ± 0.17	7.29 ± 0.95	0.660
eGFR (mL/min/1.73 m^2^)	9.73 ± 0.55	9.01 ± 0.96	0.830
Urea (mg/dL)	82.2 ± 2.55	75.65 ± 17.07	0.536
Hct (%)	35.83 ^a^ ± 0.29	32.15 ^b^ ± 1.82	**0.038**
Hb (g/dL)	11.93 ^a^ ± 0.1	10.53 ^b^ ± 0.6	**0.027**
Hb/Creatinine ratio	1.91 ± 0.04	1.67 ± 0.29	0.208
RBC (cells/μL)	3.85 ^a^ ± 0.03	3.43 ^b^ ± 0.21	**0.050**
WBC (cells/μL)	6.68 ± 0.11	7.29 ± 0.6	0.313
LYMPH (cells/μL)	1.66 ± 0.04	1.61 ± 0.24	0.850
PLT (cells/μL)	215.67 ± 4.04	207.63 ± 13.05	0.959
Albumin (g/dL)	4.31 ± 0.03	4.3 ± 0.17	0.736
Total protein (g/dL)	7.1 ± 0.05	7.15 ± 0.32	0.800
Diuresis (mL/per day)	631.09 ± 43.69	481.25 ± 210.85	0.425

The bolded values are significant at *p* < 0.05. The different letters indicate statistically significant differences (*p* ≤ 0.05).

**Table 4:** Comparison of the measured parameters in BKPyV-DNAemia-negative patients with the group with high BKPyV replication in blood (BKPvV-DNAemia viral load > 1 × 10^4^) before RTx in the A0 period.

Parameter	BKPyV-DNAemia-Negative—n = 238	BKPvV-DNAemia	*p*-Value
		Viral Load > 1 × 10^4^ n = 5	
CRP (mg/L)	2.62 ± 0.16	2.24 ± 1.09	0.606
D-dimer (ng/mL)	854.95 ± 87.33	356.67 ± 17.37	0.216
Na^+^ (mmol/L)	139.39 ± 0.21	138.2 ± 0.97	0.229
K^+^ (mmol/L)	4.8 ± 0.04	5.11 ± 0.22	0.187
Creatinine (mg/dL)	6.94 ± 0.17	6.72 ± 1.46	0.550
eGFR (mL/min/1.73 m^2^)	9.73 ± 0.55	9.7 ± 1.43	0.657
Urea (mg/dL)	82.2 ± 2.55	81.74 ± 15.13	0.951
Hct (%)	35.83 ± 0.29	34.48 ± 1.8	0.373
Hb (g/dL)	11.93 ± 0.1	11.06 ± 0.64	0.182
Hb/Creatinine ratio	1.91 ± 0.04	1.95 ± 0.4	0.953
RBC (cells/μL)	3.85 ± 0.03	3.65 ± 0.22	0.399
WBC (cells/μL)	6.68 ± 0.11	7.31 ± 0.68	0.371
LYMPH (cells/μL)	1.66 ± 0.04	1.65 ± 0.51	0.861
PLT (cells/μL)	215.67 ± 4.04	222 ± 13.32	0.564
Albumin (g/dL)	4.31 ± 0.03	4.21 ± 0.23	0.992
Total protein (g/dL)	7.1 ± 0.05	7.24 ± 0.5	0.873
Diuresis (mL/per day)	631.09 ± 43.69	570 ± 293.09	0.688

**Table 5:** Comparison of the measured parameters in patients with DNAuria-BKPyV depending on the BKPyV genotype before RTx in the A0 period.

Parameter	DNAuria-BKPyV-Negative—n = 132	Genotype I—n = 65	Genotype IV—n = 44	*p*-Value
CRP (mg/L)	2.66 ^ab^ ± 0.24	2.88 ^a^ ± 0.26	2.04 ^b^ ± 0.32	**0.045**
D-dimer (ng/mL)	790.87 ± 97.33	819.72 ± 123.68	1041.84 ± 326.3	0.947
Na^+^ (mmol/L)	139.58 ± 0.26	138.71 ± 0.44	139.65 ± 0.49	0.333
K^+^ (mmol/L)	4.77 ± 0.06	4.87 ± 0.09	4.77 ± 0.08	0.597
Creatinine (mg/dL)	6.79 ± 0.22	7.34 ± 0.35	7.05 ± 0.33	0.408
eGFR (mL/min/1.73 m^2^)	10.15 ± 0.97	9.19 ± 0.36	9.22 ± 0.37	0.984
Urea (mg/dL)	81.85 ± 3.27	83.02 ± 4.55	82.16 ± 7.31	0.775
Hct (%)	35.49 ± 0.39	36.19 ± 0.62	35.22 ± 0.62	0.190
Hb (g/dL)	11.79 ^ab^ ± 0.13	12.21 ^a^ ± 0.19	11.52 ^b^ ± 0.23	**0.013**
Hb/Creatinine ratio	1.94 ± 0.06	1.89 ± 0.08	1.79 ± 0.09	0.441
RBC (cells/μL)	3.84 ± 0.04	3.9 ± 0.07	3.72 ± 0.07	0.127
WBC (cells/μL)	6.6 ± 0.15	6.75 ± 0.2	6.97 ± 0.24	0.461
LYMPH (cells/μL)	1.73 ± 0.06	1.55 ± 0.08	1.62 ± 0.08	0.123
PLT (cells/μL)	221.78 ± 5.8	211.03 ± 7.3	207.82 ± 7.07	0.462
Albumin (g/dL)	4.32 ± 0.04	4.27 ± 0.06	4.38 ± 0.08	0.668
Total protein (g/dL)	7.11 ± 0.06	7.06 ± 0.09	7.18 ± 0.13	0.825
Diuresis (mL/per day)	746.21 ± 67.4	482.31 ± 59.52	452.27 ± 73.89	0.102

The bolded values are significant at *p* < 0.05. The different letters indicate statistically significant differences (*p* ≤ 0.05).

**Table 6:** Genotype distribution in patients with DNAuria-BKPyV in individual study periods.

Genotype/Variant		A0—n (%)	Aw—n (%)	B—n (%)	C—n (%)	D—n (%)
BKPyV-Negative		132 (53.66)	141 (57.79)	85 (49.42)	84 (51.22)	90 (53.57)
BKPyV-Positive		114 (46.34)	103 (42.21)	87 (50.58)	80 (48.78)	78 (46.43)
Genotype I		65 (57.02)	58 (56.31)	54 (62.07)	48 (60.00)	48 (61.54)
	Ib-2_POL_K	38 (58.46)	37 (63.79)	33 (61.11)	29 (60.42)	28 (58.33)
	Ib-2_POL_F	27 (41.54)	21 (36.21)	21 (38.89)	19 (39.58)	20 (41.67)
Genotype IV		44 (38.60)	40 (38.83)	29 (33.33)	28 (35.00)	26 (33.33)
Co-infection		5 (4.38)	5 (4.86)	4 (4.60)	4 (5.00)	4 (5.13)

A0—before RTx, Aw—1 month after RTx, B—3 months after RTx, C—6 months after RTx, D—12 months after RTx.

**Table 7:** Genotype distribution in patients with BKPvV-DNAemia in individual study periods.

Genotype/Variant		A0—n (%)	Aw—n (%)	B—n (%)	C—n (%)	D—n (%)
BKPyV-Negative		238 (96.75)	217 (88.93)	127 (73.84)	125 (76.22)	135 (80.36)
BKPyV-Positive		8 (3.25)	27 (11.07)	45 (26.16)	39 (23.78)	33 (19.64)
Genotype I		3 (37.50)	13 (48.15)	25 (55.56)	20 (51.28)	15 (45.45)
	Ib-2_POL_K	3 (100)	11 (84.62)	19 (76.00)	14 (70.00)	10 (66.67)
	Ib-2_POL_F	0 (0)	2 (15.38)	6 (24.00)	6 (30.00)	5 (33.33)
Genotype IV		4 (50.00)	11 (40.74)	16 (35.56)	16 (41.03)	14 (42.42)
Co-infection		1 (12.50)	3 (11.11)	4 (8.89)	3 (7.69)	4 (12.13)

A0—before RTx, Aw—1 month after RTx, B—3 months after RTx, C—6 months after RTx, D—12 months after RTx.

**Table 8:** Viral load (copies/mL) depending on isolated genotypes and variants in patients with DNAuria-BKPyV in individual study periods.

DNAuria-BKPyV						
Genotype/Variant		A0	Aw	B	C	D
BKPyV-Positive		4.21 × 10^6^	7.64 × 10^7^	4.10 × 10^9^	3.54 × 10^9^	3.76 × 10^8^
Genotype I		7.27 × 10^6^	1.29 × 10^8^	6.48 × 10^9^	3.39 × 10^8^	4.12 × 10^8^
	Ib-2_POL_K	1.24 × 10^7^	2.01 × 10^8^	8.49 × 10^9^	4.53 × 10^8^	6.42 × 10^8^
	Ib-2_POL_F	2.16 × 10^4^	2.48 × 10^6^	3.32 × 10^9^	1.64 × 10^8^	9.03 × 10^7^
Genotype IV		1.20 × 10^5^	9.54 × 10^6^	2.46 × 10^8^	9.54 × 10^9^	3.66 × 10^8^
Co-infection		3.49 × 10^5^	1.41 × 10^6^	2.22 × 10^5^	4.07 × 10^5^	3.57 × 10^5^

A0—before RTx, Aw—1 month after RTx, B—3 months after RTx, C—6 months after RTx, D—12 months after RTx.

**Table 9:** Viral load (copies/mL) depending on isolated genotypes and variants in patients with BKPvV-DNAemia in individual study periods.

BKPyV-DNAemia						
Genotype/Variant		A0	Aw	B	C	D
BKPyV-Positive		3.10 × 10^4^	3.68 × 10^5^	5.71 × 10^5^	1.15 × 10^7^	3.85 × 10^6^
Genotype I		5.75 × 10^4^	4.59 × 10^5^	8.17 × 10^5^	2.21 × 10^7^	4.85 × 10^6^
	Ib-2_POL_K	5.75 × 10^4^	5.42 × 10^5^	1.06 × 10^6^	3.15 × 10^7^	7.24 × 10^6^
	Ib-2_POL_F	Non detected	2.40 × 10^3^	4.00 × 10^4^	1.00 × 10^5^	6.26 × 10^4^
Genotype IV		1.12 × 10^4^	3.57 × 10^5^	3.27 × 10^5^	4.67 × 10^5^	3.88 × 10^6^
Co-infection		3.10 × 10^4^	1.02 × 10^4^	9.23 × 10^3^	6.00 × 10^3^	2.57 × 10^3^

A0—before RTx, Aw—1 month after RTx, B—3 months after RTx, C—6 months after RTx, D—12 months after RTx.

**Table 10:** Comparison of the measured parameters in BKPyV-DNAemia-negative patients with the group with high BKPyV replication in blood (BKPvV-DNAemia viral load > 1 × 10^4^) after RTx in period D.

Parameter	BKPyV-DNAemia-Negative n = 135	BKPvV-DNAemia	*p*-Value
		Viral Load > 1 × 10^4^ n = 20	
CRP (mg/L)	4.01 ± 1.28	12.32 ± 5.78	0.064
D-dimer (ng/mL)	709.54 ^a^ ± 90.32	1251.68 ^b^ ± 320.35	**0.018**
Na^+^ (mmol/L)	141.79 ± 0.25	141.67 ± 0.66	0.835
K^+^ (mmol/L)	4.43 ± 0.04	4.47 ± 0.13	0.989
Creatinine (mg/dL)	1.30 ^a^ ± 0.04	2.87 ^b^ ± 0.46	**<0.001**
eGFR (mL/min/1.73 m^2^)	63.99 ^a^ ± 1.64	34.84 ^b^ ± 4.74	**<0.001**
Urea (mg/dL)	50.07 ^a^ ± 1.58	90.90 ^b^ ± 11.8	**<0.001**
Hct (%)	42.32 ^a^ ± 0.48	37.69 ^b^ ± 1.59	**0.002**
Hb (g/dL)	13.84 ^a^ ± 0.15	12.32 ^b^ ± 0.56	**0.003**
Hb/Creatinine ratio	11.83 ^a^ ± 0.35	6.25 ^b^ ± 0.88	**<0.001**
RBC (cells/μL)	4.76 ^a^ ± 0.06	4.10 ^b^ ± 0.18	**0.001**
WBC (cells/μL)	7.26 ^a^ ± 0.18	7.01 ^b^ ± 0.92	**0.034**
LYMPH (cells/μL)	1.96 ± 0.06	2.01 ± 0.39	0.231
PLT (cells/μL)	236.98 ± 7.03	238.2 ± 23.37	0.574
Albumin (g/dL)	4.38 ± 0.04	4.24 ± 0.17	0.592
Total protein (g/dL)	7.06 ± 0.05	6.82 ± 0.2	0.258
Diuresis (mL/per day)	2616.67 ^a^ ± 53.23	2080 ^b^ ± 117.34	**<0.001**

The bolded values are significant at *p* < 0.05. The different letters indicate statistically significant differences (*p* ≤ 0.05).

**Table 11:** Characteristics of the study group before RTx—period A0.

Variables	Mean ± SEM or n (%)
Female n (%)	83 (33.74)
Male n (%)	163 (66.26)
Height (m)	1.71 ± 0.006
Weight (kg)	72.18 ± 0.90
BMI (kg/m^2^)	24.61 ± 0.24
Blood group n (%)	
A	103 (41.87)
B	40 (16.26)
AB	23 (9.35)
0	80 (32.52)
Age of (years)	
Renal disease diagnosis	35.07 ± 1.00
Progression to ESRD (months)	95.54 ± 6.38
ESRD	43.17 ± 0.91
Dialysis time (months)	38.42 ± 1.95
Transplantation	47.11 ± 0.84
ESRD etiology, n (%)	
Glomerular	101 (41.06)
Vascular or diabetes	55 (22.36)
Genetic	46 (18.7)
Obstructive	19 (7.72)
Interstitial or other	25 (10.16)
Diuresis (mL/day) and dialysis (n, %)	
Diuresis	626.22 ± 42.78
Diuresis < 500 mL/day	123 (50.0)
CADO	48 (19.51)
CADO—diuresis	730.83 ± 112.29
CADO—diuresis < 500 mL/day	21 (43.75)
HD	197 (80.08)
HD—diuresis	596.29 ± 45.63
HD—diuresis < 500 mL/day	102 (51.78)
Pre-emptive	1 (0.41)
Pre-emptive—diuresis	1500

**Table 12:** Characteristics of the DNAuria-BKPyV group.

Variables	DNAuria-BKPyV—Positive	DNAuria-BKPyV—Negative	*p*-Value	Total—n (%)
	n—114 (%)	n—132 (%)		
Gender				
Female	37 (32.46)	46 (34.85)	0.69	83 (33.74)
Male	77 (67.54)	86 (65.15)		163 (66.26)
Age (years)				
18–29	10 (8.77)	15 (11.36)	0.54	25 (10.16)
30–39	26 (22.81)	31 (23.48)		57 (23.17)
40–49	25 (21.93)	28 (21.21)		53 (21.54)
50–59	30 (26.32)	31 (23.48)		61 (24.8)
60–69	21 (18.42)	19 (14.39)		40 (16.26)
>70	2 (1.75)	8 (6.06)		10 (4.07)
BMI (kg/m^2^) n (%)				
<18.49	7 (6.14)	7 (5.3)	0.94	14 (5.69)
18.5–24.99	58 (50.88)	65 (49.24)		123 (50)
>25	49 (42.98)	60 (45.45)		109 (44.31)
Blood group n (%)				
A	47 (41.23)	56 (42.42)	0.24	103 (41.87)
B	19 (16.67)	21 (15.91)		40 (16.26)
AB	15 (13.16)	8 (6.06)		23 (9.35)
0	33 (28.95)	47 (35.61)		80 (32.52)
Diuresis n (%)				
<500 mL/d	62 (54.39)	61 (46.21)	0.20	123 (50)
>500 mL/d	52 (45.61)	71 (53.79)		123 (50)
ESRD etiology n (%)				
Glomerular	50 (43.86)	51 (38.64)	0.65	101 (41.06)
Vascular or diabetes	25 (21.93)	30 (22.73)		55 (22.36)
Genetic	23 (20.18)	23 (17.42)		46 (18.7)
Obstructive	7 (6.14)	12 (9.09)		19 (7.72)
Interstitial or other	9 (7.89)	16 (12.12)		25 (10.16)
Immunosuppression n (%)				
CNIs	102 (89.47)	132 (100)	0.48	234 (95.12)
Mycophenolate	110 (96.49)	132 (100)		242 (99.19)
Steroids	114 (100)	132 (100)		246 (100)
Everolimus	12 (10.53)	0 (0)		12 (4.88)
Induction n (%)				
Basiliximab	62 (54.39)	70 (53.03)	0.16	132 (53.66)
ATG	4 (3.51)	7 (5.30)		11 (4.47)
No induction	48 (42.11)	55 (41.67)		103 (41.87)
Complications n (%)				
DGF	47 (41.23)	67 (58.77)	0.23	114 (46.34)
AR	27 (23.68)	20 (15.15)		47 (19.11)
Biopsy	37 (32.46)	30 (22.73)		67 (27.24)
BKPyVAN	3 (2.63)	0 (0)		3 (1.22)
Death	2 (1.75)	2 (1.52)		4 (1.63)
Total	114 (100)	132 (100)		246 (100)

## Data Availability

The original contributions presented in this study are included in the article and Supplementary Materials. Further inquiries can be directed to the corresponding authors.

## Supplementary Materials

The following supporting information can be downloaded at: https://www.mdpi.com/article/10.3390/ijms27062832/s1.
