# Supplementary material for: BKPyV—Co-Architect of the Fate of a Renal Transplant During a One-Year Observation Period
Source: Int J Mol Sci. 2026 Mar 20;27(6):2832. doi: 10.3390/ijms27062832 (PMC13027097; doi:10.3390/ijms27062832)
Supplement: Supplementary file 1 [file ijms-27-02832-s001.zip › ijms-4188756-supplementary/Figures S1, S2 and S3.pdf]

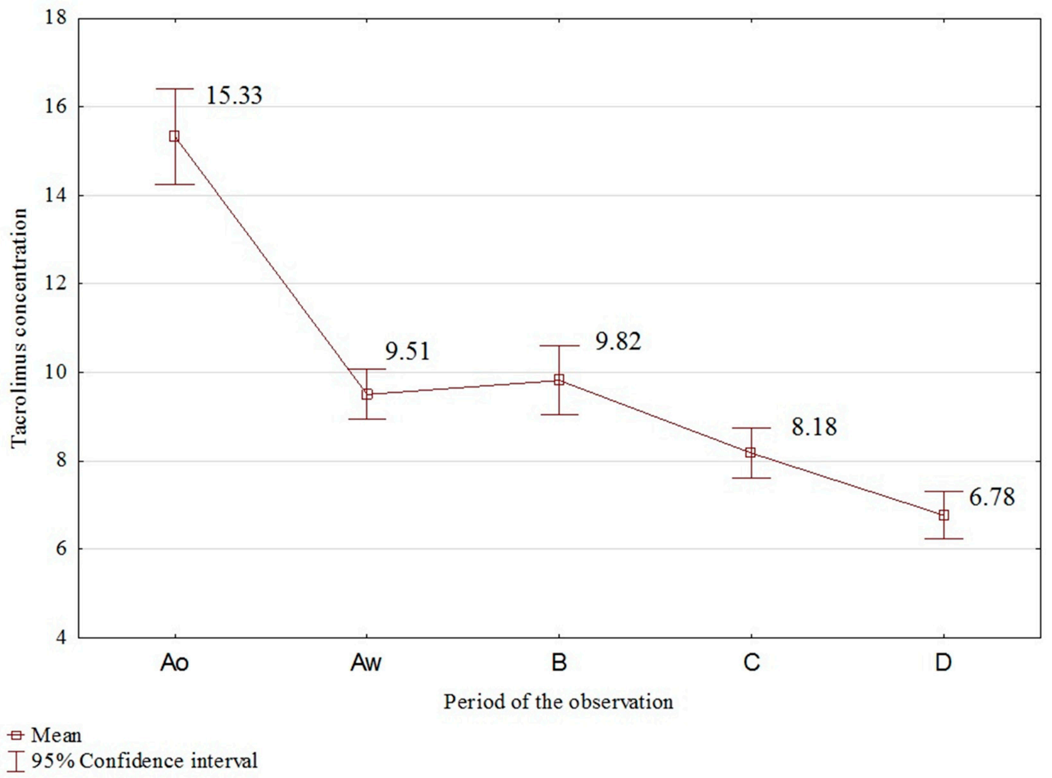

Figure S1. Immunosuppressive treatment – Tacrolimus concentration over time of observation.

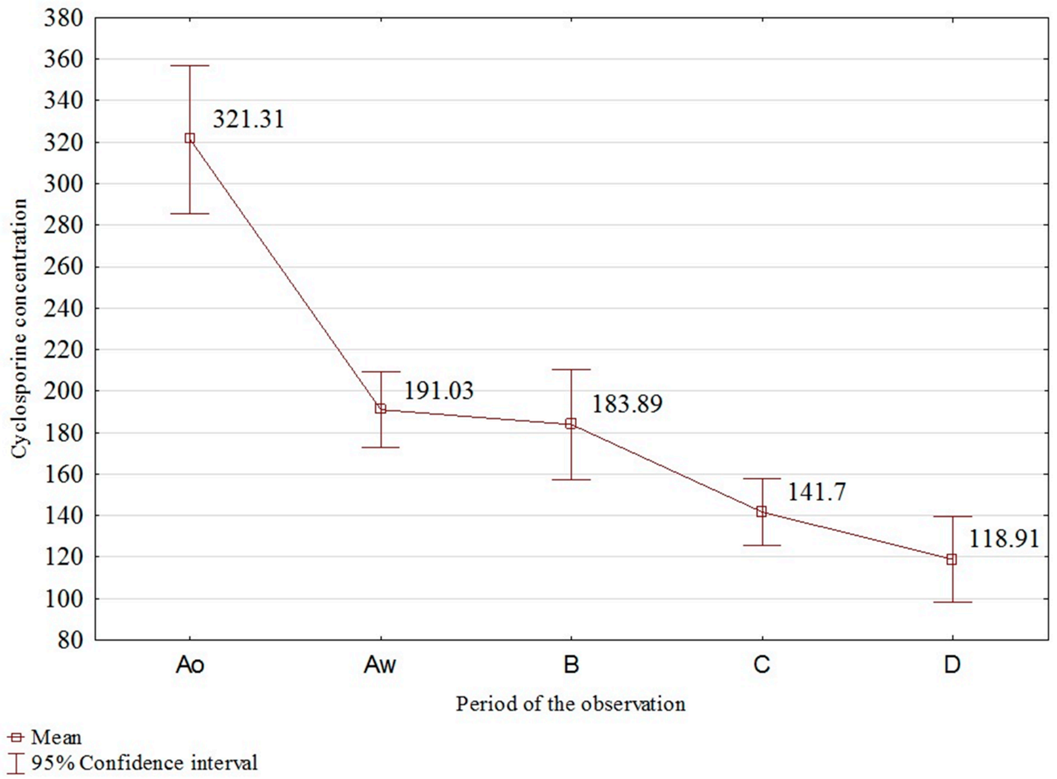

Figure S2. Immunosuppressive treatment – Cyclosporine concentration over time of observation.

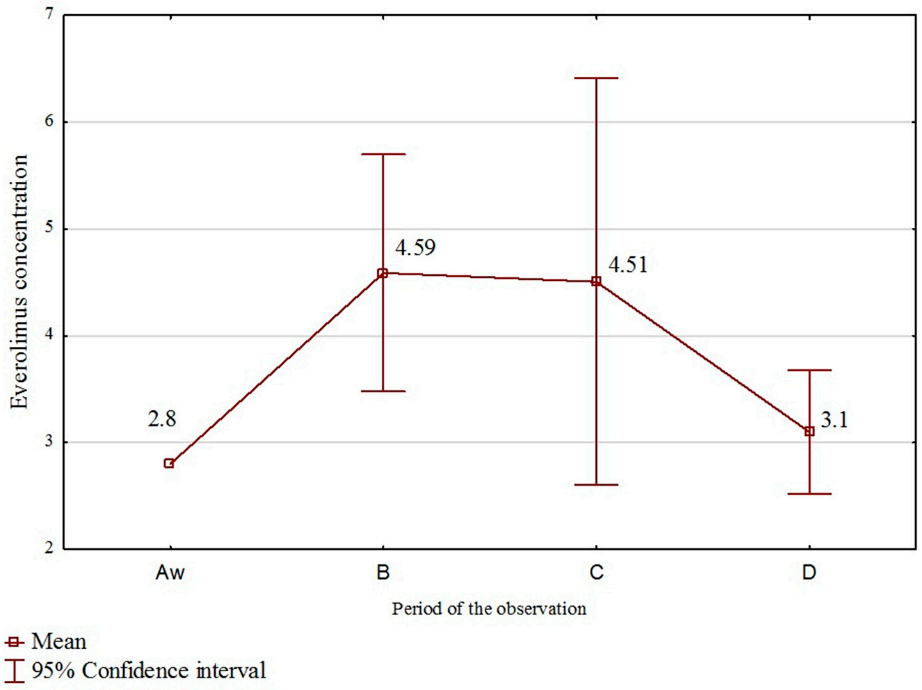

Figure S3. Immunosuppressive treatment – Everolimus concentration over time of observation.
